# The contribution of biophysical and biochemical CO_2_ concentration mechanisms to the carbon fixation of the green macroalga *Ulva prolifera*

**DOI:** 10.1007/s42995-024-00265-7

**Published:** 2024-12-12

**Authors:** Xiaohua Zhang, Guang Gao, Zhengquan Gao, Kunshan Gao, Dongyan Liu

**Affiliations:** 1 School of Pharmacy, Binzhou Medical University, Yantai, 264003 China; 2 State Key Laboratory of Marine Environmental Science, Xiamen University (Xiang’an Campus), Xiamen, 361102 China; 3 State Key Laboratory of Estuarine and Coastal Research, East China Normal University, Shanghai, 200241 China

**Keywords:** CO_2_ concentration mechanism, Photosynthesis, Carbon fixation, Carbonic anhydrase, C_4_ metabolism

## Abstract

CO_2_ concentration mechanisms (CCMs) are important in maintaining the high efficiency of photosynthesis of marine algae. Aquatic photoautotrophs have two types of CCMs: biophysical CCMs, based on the conversion of inorganic carbon, and biochemical CCMs, based on the formation of C_4_ acid intermediates. However, the contribution of biophysical and biochemical CCMs to algal carbon fixation remains unclear. Here, we used ethoxyzolamide (EZ) inhibitors of carbonic anhydrase and 3-mercaptopicolinic acid (MPA) inhibitors for phosphoenolpyruvate carboxykinase to examine the importance of biophysical and biochemical CCMs in photosynthesis of the green macroalga *Ulva prolifera*. The culture experiments showed that the carbon fixation of the species declined when EZ inhibited the biophysical CCM, while the increase in cyclic electron flow around the photosystem I indicated a more active biochemical CCM, contributing to ~ 50% of total carbon fixation. The biophysical CCM was also reinforced when MPA inhibited the biochemical CCM. In a comparison, the biophysical CCM can compensate for almost 100% of total carbon fixation. The results indicate that biophysical CCMs dominate the process of carbon fixation of *U. prolifera* while biochemical CCM plays a supporting role. Our results provide evidence of a complementary coordination mechanism between the biophysical and biochemical CCMs that promotes the efficiency of photosynthesis of *U. prolifera*, an efficient mechanism to boost the alga’s bloom.

**Supplementary Information:**

The online version contains supplementary material available at 10.1007/s42995-024-00265-7.

## Introduction

In aquatic environment, the CO_2_ concentration mechanisms (CCMs) play a vital role in promoting the efficiency of algal photosynthesis, because of the slow diffusion of CO_2_ in water and the low affinity of ribulose-1,5-bisphosphate carboxylase/oxygenase (RuBisCO) for CO_2_ (Beardall and Raven 2020). CCMs can be divided into biophysical CCMs and biochemical CCMs, based on either biophysical processes or biochemical mechanisms: In biochemical CCMs, inorganic carbon is first fixed to an intermediate form of organic carbon before finally being decarboxylated to produce CO_2_ for RubisCO. A biophysical CCM is an “inorganic” CCM that does not rely on additional organic carbon intermediates, but instead increases the CO_2_ concentration near RubisCO by interconversion of inorganic carbon forms (Beardall and Raven 2020; Clement et al. 2016; Maberly and Gontero 2017; Reinfelder 2011). Biophysical CCMs, which support the active transport of Ci via the actions of carbonic anhydrases (CA) and bicarbonate transporters, have been well documented in algal photosynthesis (Beer 2022; Moroney et al. 2011; Wang et al. 2015). CA either catalyzes the dehydration of HCO_3_^−^ to produce CO_2_ at the cell surface, which then diffuses into the cell, or converts HCO_3_^−^ to CO_2_ intracellularly (Bowes. 1969; Gee and Niyogi 2017; Jensen et al. 2019). For some algae, e.g., the diatom *Thalassiosira weissflogii* and the green alga *Udotea flabellum*, they not only have a biophysical CCM but also a biochemical CCM; the latter is defined as one where an initial fixation of Ci transports into C_4_ acid supporting C_4_ photosynthesis (Koch et al. 2013; Reinfelder et al. 2000; Reiskind and Bowes 1991). In diatom C_4_ photosynthesis, HCO_3_^−^ transported into the cytoplasm is fixed to C_4_ acid by phosphoenolpyruvate carboxylase (PEPC), then the products transported to chloroplasts, decarboxylated by phosphoenolpyruvate carboxykinase (PEPCK) to produce CO_2_ (Reinfelder et al. 2000). The active C_4_ key enzymes, formation of C_4_ acids, and decarboxylation of C_4_ acids are considered key features of a typical C_4_ system (Johnston et al. 2001).

Environmental variations can coordinate the function of the two different CCMs and determine their contributions to carbon fixation in algal photosynthesis (Raven et al. 2017). For example, the combination of biochemical and biophysical CCMs in *T. weissflogii* confers plasticity in acclimating to changing environments, where the biochemical CCM contributes the major role in Zn-stressed environments (Reinfelder et al. 2000; Roberts et al. 2007). The relative importance of biophysical versus biochemical CCMs in algal carbon fixation is the focus of current research (Reinfelder 2011). Although CCMs in phytoplankton are active under diverse environmental conditions, it is difficult to distinguish whether a biophysical or a biochemical CCM is operating, and thus the relative contributions of biochemical and biophysical CCMs to carbon fixation remain controversial (Clement et al. 2016; Haimovich-Dayan et al. 2013). Inhibition of the corresponding CCMs by inhibitors, such as the PEPCK inhibitor 3-mercaptopicolinic acid (MPA) to inhibit the biochemical CCM (McGinn et al. 2008; Reiskind and Bowes 1991), or the CA inhibitors acetazolamide (AZ) and ethoxyzolamide (EZ) to inhibit the biophysical CCM (Zuñiga-Rios et al. 2021), can be used to assess the relative contributions of each CCM.

*Ulva prolifera,* the main species causing green tides in the Yellow Sea, grows rapidly and can increase its biomass by up to 37% per day (Hiraoka et al. 2020; Cui et al. 2015; Liu et al. 2013). *U. prolifera* is able to tolerate harsh environmental conditions, such as high irradiance, and eventually covers an area of over 20 000 km^2^ in the Yellow Sea, suggesting efficient photosynthesis (Cui et al. 2015; Zhang et al. 2019). Previous studies have found that the efficient photosynthesis and carbon fixation of *U. prolifera* is related to its diverse CCMs and C_4_ pathway, e.g., the C_4_-related genes, enzymes, and carbon products were examined (Liu et al. 2020; Valiela et al. 2018; Xu et al. 2012). These data provide circumstantial evidence for C_4_-like photosynthesis, but do not establish a direct correlation between C_4_ acid and carbon fixation. *U. prolifera* appears to regulate the different CCMs in response to changing environmental conditions. For example, the activities of C_4_ key enzymes such as PEPC, PEPCK or pyruvate orthophosphate dikinase are more active under stress conditions (Gu et al. 2022; Liu et al. 2020; Xu et al. 2012; Zhao et al. 2023), while the expression of CA-encoding genes was sensitive to a range of environmental factors (Wang et al. 2020, 2021). It is speculated that the biophysical and biochemical CCMs in photosynthetic carbon fixation of *U. prolifera* should be coordinated by environmental conditions. Therefore, the importance of biochemical CCM in the carbon fixation of *U. prolifera* requires more definitive evidence, such as the role of C_4_ acids in photosynthetic carbon fixation; the individual contribution of biophysical and biochemical CCMs in photosynthetic carbon fixation.

In this study, an attempt was made to confirm the importance of biochemical CCMs in *U. prolifera* photosynthesis by measuring the effects of C_4_ acid on photosynthetic O_2_ evolution and inorganic carbon fixation. The contribution of biophysical and biochemical CCMs under fluctuating light conditions was estimated by adding two inhibitors (EZ for CA and MPA for PEPCK) to culture experiments, respectively, to regulate photosynthetic carbon fixation.

## Materials and methods

### Materials

*Ulva prolifera* samples were collected from the coastal water of Qingdao (36.09°N, 120.50°E), Shandong province, China. Healthy algal thalli were selected, gently washed to remove contaminants, then incubated in sterile seawater containing 0.1 g/L neomycin sulfate, 0.03 g/L polymyxin B and 0.1 g/L penicillin G. The antibiotic medium was changed every 3–5 days until no bacteria could be detected (Chen et al. 2019). Thalli samples were then sub-cultured in antibiotic-free f/2 medium at 22 °C with 50 μmol photons m^−2^ s^−1^ (Light: Dark = 12 h/12 h) in a laboratory incubator (HZ100LG, Ruihua, Wuhan, China).

### Photosynthetic oxygen evolution measurements

Photosynthetic O_2_ evolution was determined using a Clark-type O_2_ electrode system (Hansatech, King’s Lynn, UK) at 22 °C and 200 μmol photons m^−2^ s^−1^ quantum irradiance. The *U. prolifera* samples were cut into 1-cm-long fragments with scissors and then cultured in natural seawater for more than 2 h before O_2_ evolution measurements were made. Prior to measurement, the fragments were transferred to buffered artificial seawater (20 mmol/L Hepes–NaOH, pH 8.0) in the absence of Ci for 30 min to deplete endogenous Ci sources; the buffered artificial seawater was previously aerated at low pH with pure N_2_ to remove CO_2_ (Berges et al. 2001; Gao. 1999; Li et al. 2016).

### CA and PEPCK inhibition experiments

AZ (A832214, Macklin reagent), a specific inhibitor of external periplasmic CA, and EZ (333,328, Sigma-Aldrich), an inhibitor of total CA activity (extracellular and intracellular) were used to inhibit external CA and internal CA activity, respectively (Björk et al. 1992). MPA (SC-206655, Santa Cruz Biotechnology) was used to inhibit the PEPCK activity (Reiskind and Bowes 1991). For inhibition treatments, inhibitors were added to the buffered artificial seawater with 2 mmol/L NaHCO_3_, and the photosynthetic O_2_ rates were measured. Through gradient concentration detection, a final concentration of 50 µmol/L EZ and 1.5 mmol/L MPA was added to the medium for subsequent photosynthetic O_2_ evolution and enzyme activity assays. The percentage inhibition of the photosynthetic O_2_ evolution by inhibitors was calculated by the formula 100 x [1—(rate with inhibitors) / (rate without inhibitors)].

### C_4_ acid-dependent O_2_ evolution experiments

The importance of C_4_ organic carbon in *U. prolifera* photosynthesis was tested by measuring the effects of C_4_ compounds on photosynthetic O_2_ evolution. In this experiment, whether the C_4_ compounds (oxaloacetic acid (OAA) and aspartic acid (Asp)) support photosynthetic O_2_ evolution in the absence of exogenous Ci in an O_2_ electrode system was investigated. OAA (O4126, Sigma-Aldrich) or Asp (A9256, Sigma-Aldrich) was added to the buffered artificial seawater, which was sparged with CO_2_-free air at low pH, with or without inhibitors (50 µmol/L EZ or 1.5 mmol/L MPA, respectively). Photosynthetic rates of OAA-added thalli with ambient CO_2_ (400 ppm) and high CO_2_ (1000 ppm) were also measured. All experiments were performed in three or four replicates.

### C_4_-Ci carbon fixation competition experiment

To measure the effect of OAA on inorganic carbon fixation, *U. prolifera* was incubated with or without 2 mmol/L OAA in buffered artificial seawater (20 mmol/L Hepes–NaOH, pH 8.0) containing 2 mmol/L NaH^13^CO_3_ (CLM-441–5, Cambridge Isotope Laboratories) for 2 h at 22 °C. At the start of the incubation, the original atom % of ^13^C was determined. The samples were washed with 1 N HCl and exposed to fuming HCl for 2 h to remove carbohydrates, then dried at 50 °C for 12 h for the isotopic analysis. Approximately 0.5 mg samples were taken for the particulate organic carbon and the isotopic ratio of ^13^C to ^12^C determination using an elemental analyzer (EA; Vario PYRO Cube, Elementar, Germany) coupled to a continuous flow isotope ratio mass spectrophotometer (Delta V Advantage, Thermo Scientific, Germany). The photosynthetic production (*Δ*C) was calculated according to Hama et al. (1983) and expressed in µg ^13^C /mg dry weight. The difference in *Δ*C (µg ^13^C /mg dry weight) between the group with and without OAA was estimated to derive from OAA.

### Enzyme activity assays

The PEPC and PEPCK activities were assayed using enzyme assay kits provided by Solarbio Life Sciences, China. To determine PEPC and PEPCK activities in response to EZ, *U. prolifera* was cultured in buffered artificial seawater (as described above) containing 2 mmol/L NaHCO_3_ in the presence of 50 µmol/L EZ for 4 h and sampled at regular intervals for PEPC and PEPCK activity assays. To determine PEPC and PEPCK activities in response to light intensities, *U. prolifera* was cultured under low light (200 μmol photons m^−2^ s^−1^) or high light (1300 μmol photons m^−2^ s^−1^) with 2 mmol/L NaHCO_3_ for 2 h. During the culture, *U. prolifera* was placed in the sealed quartz tube at 22 °C and illuminated by a simulated solar radiator. All assays were performed in three or four replicates.

### Outdoor experiments

The *U. prolifera* was cut into pieces and cultured overnight in natural seawater prior to the outdoor experiment to minimize damage by sampling cuts. Approximately 8 g of fresh thalli were placed into a 1 L quartz tubes with 0.5 L of sterile buffered artificial seawater with f/2 medium (without Si), 2 mmol/L NaH^13^CO_3_ was added as the sole carbon source in each group. The experiment was set up in three groups, according to the inhibitors: control group (without any inhibitors); MPA inhabitation group (the thalli were incubated with 2 mmol/L MPA for 30 min prior to the addition of NaH^13^CO_3_); and EZ inhabitation group (simultaneously add 2 mmol/L NaH^13^CO_3_ and 100 μmol/L EZ). Each group had three replicates. All the quartz tubes were suspended in an outdoor pool at Xiang’ An campus of Xiamen University, China. Water temperature,which was maintained at 33.4 – 34.0 °C, was monitored during the culture using a thermometer; solar irradiance was continuously monitored using a filter radiometer, located on the roof of the Zhou Longquan building, about 2 km from the experimental site. The experiment lasted 12 h, from 08:00 to 18:00, samples were collected from each of the tubes every 2 h and stored in a liquid nitrogen tank for the subsequent assays.

### Enzyme activities

About 0.1 g of each frozen sample was ground on ice using an electric tissue grinder for the PEPC, PEPCK, and CA activity assays. The PEPC and PEPCK activities were measured using the Solarbio Life Sciences kit, as described above. CA activity was measured according to the method of Wilbur and Anderson (1948). Approximately 0.1 g of algae thalli was crushed with an electric tissue grinder and soaked in 5 mL barbiturate buffer (20 mmol/L, pH = 8.3). Then 3 mL of pre-cooled CO_2_ saturated water was injected, and the catalytic reaction time required to change pH from 8.3 to 7.3 was recorded as *T*_c_. The reaction process was performed in a 4 ℃ water bath. The same procedure was repeated using 5 mL of barbiturate buffer without algal thalli, and the non-catalytic time of pH change from 8.3 to 7.3 was recorded as *T*_0_. CA activity was calculated using the following equation: CA activity (U/g) = [(*T*_0_/*T*_c_ − 1) × 10]/algal weight.

### Expression levels of CA, PEPC, and PEPCK

RT-qPCR was used to determine gene expression levels in different treatment groups at different time points. Total RNA was extracted from 0.1 g of frozen sample using E.Z.N.A. Plant RNA kit (Omega), and 1 μg of total RNA from each sample was transcribed into cDNA using RevertAid First Strand cDNA Synthesis Kit, with DNase I (Thermo Scientific). The RT-qPCR was performed on a Bio-rad CFX96 real-time PCR system using a TB Green® Premix Ex Taq™ II (Tli RNaseH Plus) (Takara). The reaction took place in a 10 μL volume containing 5 μL TB Green Premix Ex Taq II, 2 μL diluted cDNA (equivalent to 200 ng RNA), 1 μL of each 10 μmol/L primer and 2 μL ddH_2_O. The qPCR procedure was as follows: 95 °C for 5 min, followed by 40 cycles of 95 °C for 30 s and 60 °C for 1 min. 18S rDNA gene was used as reference gene to normalize the target genes. The specific primers (Supplementary Table S1) for the CA, PEPC, PEPCK and 18S rDNA were designed using the Primer–BLAST (http://blast.ncbi.nlm.nih.gov/). The relative gene expression levels were calculated using 2^−ΔΔCt^ method.

### Photosynthetic parameter and P700 analyses

After dark adaptation for 10 min, the fluorescence induction curves of each sample were measured using a Dual-PAM-100 device (Walz Heinz GmbH, Effeltrich, Germany) to determine the photochemical properties of *U. Prolifera* at different time points. The performance of PSII and PSI was calculated: 1) *F*v/*F*m = (*F*m–*F*o)/*F*m; 2) NPQ = (*F*m-*F*m′)/*F*m′; 3) YII = (*F*m′-*F*)/*F*m′; 4) YI = (*P*m′-*P*)/*P*m. *F*o is the minimum fluorescence of dark-acclimated sample, *F*m is the maximum fluorescence in the presence of a pulse of saturating light, *F* corresponds to the stable fluorescence under an illuminated light before application of a saturation pulse, and *F*m′ is the maximum fluorescence of the illuminated sample under the saturating-light flash. *P*m represents the maximum P700 absorption at the fully oxidized state, *P*m′ is the maximum P700 signal induced by a combined actinic irradiation plus a saturation pulse, *P* is the absorption signal of P700 in the presence of actinic light. The electron transfer rate of PSII was calculated as ETRII = YII × PAR × 0.84 × 0.5, while the electron transfer rate of PSI was calculated as ETRI = YI × PAR × 0.84 × 0.5, where PAR is the photosynthetically active photon flux density. The PSI-CEF was calculated as ETRI–ETRII (Kono et al. 2014; Huang et al. 2016).

### Statistical analysis

Statistical comparisons were performed by one-way ANOVA using SPSS 25.0 software, and differences were considered significant when *p* value < 0.05.

## Results

### Effect of CA and PEPCK inhibitors on net photosynthetic rate of *U. prolifera*

Two CA inhibitors, AZ and EZ, were used to differentiate between external inhibition (AZ) and external + internal inhibition (EZ). The culture experiments showed that EZ inhibited the net photosynthetic (O_2_ evolution) rate of *U. prolifera*, the inhibition rate of thalli photosynthesis was about 95% when its concentration reached 50 µmol/L (Table 1). In contrast, 50 µmol/L AZ had less than 5% inhibitory effect on photosynthetic O_2_ evolution (Table 1), indicating that intracellular CA acted as a major CO_2_ concentrator in *U. prolifera*. To test the role of PEPCK in *Ulva* photosynthesis, the PEPCK-specific inhibitor MPA was added to the thalli growing in the buffered seawater containing 2 mmol/L NaHCO_3_. The presence of MPA resulted in more than 90% inhibition of photosynthetic O_2_ evolution (Table 1), indicating that inhibition of PEPCK impaired *Ulva* photosynthesis.

**Table 1:** The inhibitory effects of AZ, EZ, and MPA on net photosynthetic O_2_ evolution of *Ulva prolifera*

Groups	Exogenous inhibitor concentration (µmol/L)	Photosynthetic O_2_ evolution (µmol O_2_ mg^−1^ Chl*a* h^−1^)	Inhibition, %
AZ	0	60.92 (5.34)^a^	–
	50	59.52 (5.66)^a^	2.3
EZ	0	72.32 (7.26)^a^	–
	50	3.63 (0.80)^b^	95.0
MPA	0	51.78 (11.81)^a^	–
	1500	4.25 (3.02)^b^	91.8

Photosynthetic O_2_ evolution was measured by incubating thalli with 50 µmol/L AZ, 50 µmol/L EZ or 1.5 mmol/L MPA for more than 30 min. Each data bar is the mean of three replicate measurements. Bars are standard deviations of the mean data

Different letters indicate significant differences between different inhibitor concentrations (a/b, *p* < 0.05)

*AZ* acetazolamide, *EZ* ethoxyzolamide, *MPA* 3-mercaptopicolinic acid

### The importance of the C_4_ fixed carbon in *U. prolifera*

**Fig. 1:**
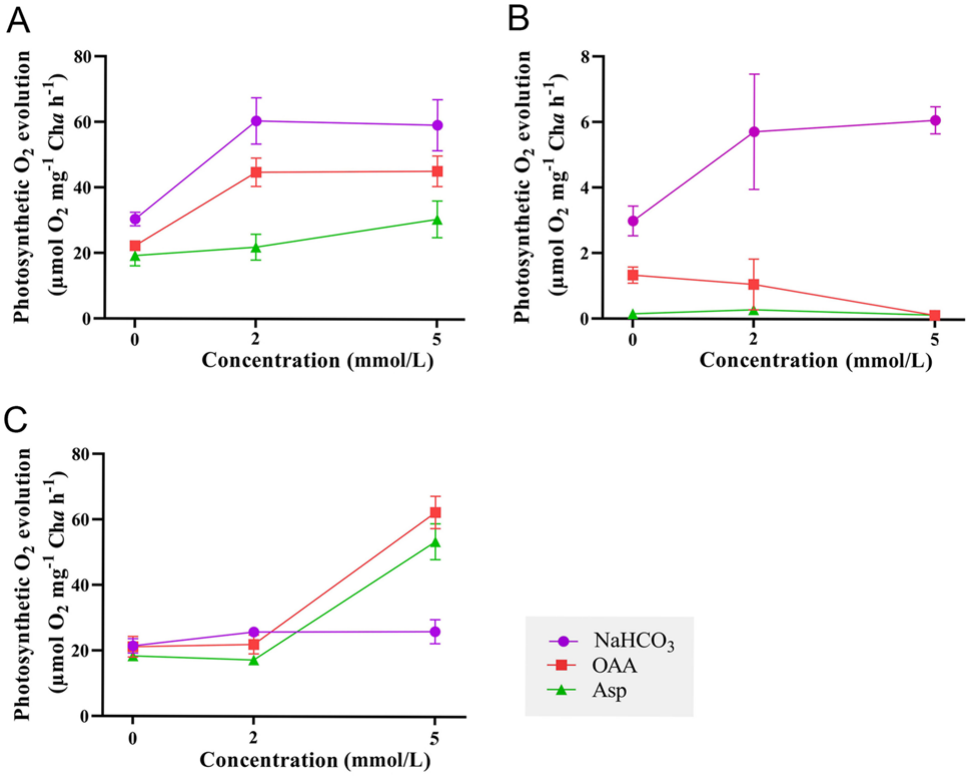
Effects of C_4_ organic acids on photosynthetic O_2_ evolution of *Ulva prolifera* in Ci-free seawater with or without inhibitors. **A** Effects of OAA and Asp on photosynthetic O_2_ evolution of *U. prolifera* in Ci-free seawater. **B** Effects of OAA and Asp on photosynthetic O_2_ evolution of *U. prolifera* in Ci-free seawater with 1.5 mmol/L MPA. **C** Effects of OAA and Asp on photosynthetic O_2_ evolution of *U. prolifera* in Ci-free seawater with 50 µmol/L EZ. Each data is the mean of three replicate determinations. Bars are standard deviations of the mean data. *OAA* oxaloacetic acid, *Asp* aspartic acid, *EZ* ethoxyzolamide, *MPA* 3-mercaptopicolinic acid

The effects of OAA and Asp on the photosynthetic O_2_ evolution of *U. prolifera* cultured in Ci-free seawater were investigated. As shown in Fig. 1A, the addition of 2 mmol/L OAA to Ci-free seawater stimulated O_2_ evolution from 22 to 45 µmol O_2_ h^−1^ mg^−1^ Chl*a*, which was similar to the addition of 2 mmol/L NaHCO_3_. Asp could also support O_2_ evolution but less effectively than OAA (Fig. 1A). Both OAA and Asp failed to stimulate O_2_ evolution in the presence of MPA (Fig. 1B), whereas 5 mmol/L OAA and Asp stimulated O_2_ evolution from 21 to 62 µmol O_2_ h^−1^ mg^−1^ Chl*a* and 18 to 54 µmol O_2_ h^−1^ mg^−1^ Chl*a*, respectively, in the presence of EZ (Fig. 1C). The effects of OAA on the Ci fixation of *U. prolifera* were further determined. As shown in Table 2, the ^13^C ratio reached 2.35% after incubation with 2 mmol/L NaH^13^CO_3_ for 2 h, whereas ^13^C incorporation was significantly reduced when OAA was added (*p* < 0.05). The addition of 2 mmol/L OAA inhibited the carbon fixation provided by NaH^13^CO_3_ by up to 55% (Table 2), suggesting that OAA could provide a significant portion of the carbon fixation for *U. prolifera*.

**Table 2:** Effects of oxaloacetic acid on the determination of ^13^C % and photosynthetic production (*Δ*C) in *Ulva prolifera*

Groups	^13^C (%)	Photosynthetic production (*Δ*C) (µg ^13^C mg^−1^ dry weight)
Original sample	1.09 (0.00)^c^	–
Control	2.29 (0.17)^a^	3.40 (0.61)^a^
Plus oxaloacetic acid	1.48 (0.23)^b^	1.51 (0.81)^b^

*U. prolifera* was pre-cultured in buffered Ci-free artificial seawater for 1 h to consume intracellular CO_2_, and then was transferred to buffered artificial seawater containing NaH^13^CO_3_ as the sole carbon source with or without 2 mmol/L OAA. After 2 h, the thalli incubated with or without OAA were gathered to measure the particulate organic carbon and incorporated ^13^C%. The *Δ*C was calculated by the method of Hama et al (1983). Values in parentheses are the standard deviations of mean data (*n* = 3), different letters represent significant differences between different groups (a/b/c, *p* < 0.05)

Table 3 shows that the OAA-dependent O_2_ evolution rate was 2.3 times higher in thalli acclimated to low CO_2_ (400 × 10^−6^) than in those cultured under high CO_2_ conditions (1000 × 10^−6^) (*p* < 0.05). However, the CO_2_ concentration to which the thalli adapted had no significant effect on the photosynthetic O_2_ evolution of Ci (*p* > 0.05). These results suggest that C_4_ carbon fixation was modulated by the CO_2_ concentration to which the thalli have been adapted and provided carbon for photosynthesis of *U. prolifera* in low CO_2_ environment.

**Table 3:** Carbon-dependent O_2_ evolution rates in *Ulva prolifera* acclimated to 400 or 1000 × 10^–3^ CO_2_

[CO_2_]	1 mmol/L Ci	1 mmol/L oxaloacetic acid
	photosynthetic O_2_ evolution (µmol O_2_ mg^−1^ Chl*a* h^−1^)	photosynthetic O_2_ evolution (µmol O_2_ mg^−1^ Chl*a* h^−1^)
400 × 10^−6^	167.48 (15.26)^a^	146.91 (21.48)^a^
1000 × 10^−6^	149.97 (9.93)^a^	78.95 (15.15)^b^

Photosynthetic O_2_ evolution was measured with either 1 mmol/L oxaloacetic acid or NaHCO_3_. Values in parentheses are the standard deviations of mean data (*n* = 4). Different letters indicate significant differences between the CO_2_ levels (a/b, *p* < 0.05)

### The modulation of C_4_ route in *U. prolifera*

**Fig. 2:**
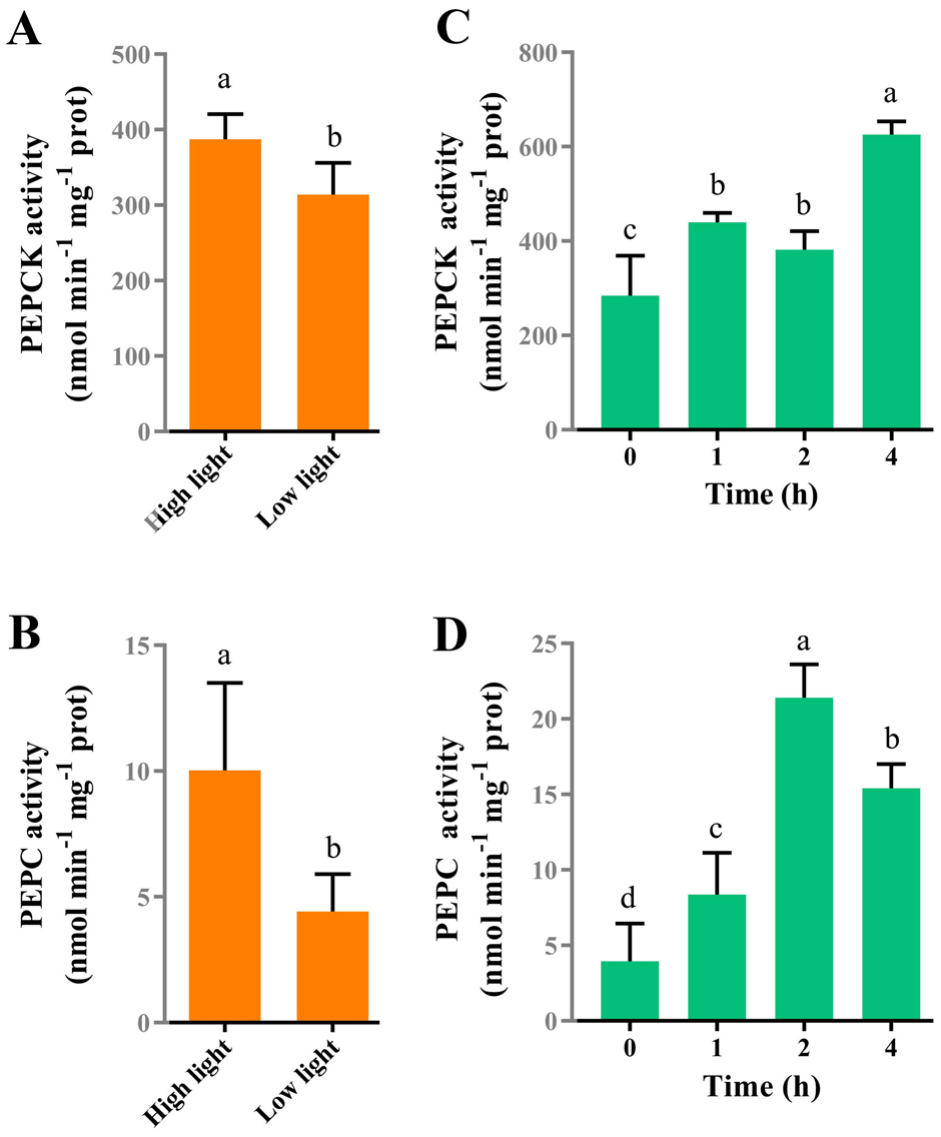
PEPCK and PEPC activities in *Ulva prolifera.* PEPCK (**A**) and PEPC (**B**) activities in response to light intensity. PEPCK (**C**) and PEPC (**D**) activities in response to carbonic anhydrase inhibitor ethoxyzolamide (50 µmol/L). All enzyme activities are the means of three or four replicate assays and bars are standard deviations of the mean data. Different letters represent significant differences between groups (a/b/c/d, *p* < 0.05). *PEPCK* phosphoenolpyruvate carboxykinase, *PEPC* phosphoenolpyruvate carboxylase

Since light intensity is an important factor affecting C_4_ pathway in plants, the effect of light intensity on PEPC and PEPCK was determined. PEPC and PEPCK showed a similar response to changes in light intensity (Fig. 2A, B): PEPC and PEPCK activities under high light conditions were significantly higher than those under low light conditions (*p* = 0.006, *p* = 0.042, respectively). As shown in Fig. 2C, D, PEPCK activity increased significantly from 284 to 626 nmol min^−1^ mg^−1^ prot after EZ treatment for 4 h (*p* < 0.05), and PEPC activity reached a maximum after 2 h of EZ treatment with a 5.4-fold increase. These results indicate that the C_4_ route was modulated by irradiance and the activity of biophysical CCM.

### The contribution of biophysical and biochemical CCMs to carbon fixation with diurnal sunlight variations

Three outdoor experiments (control group, EZ inhabitation group, and MPA inhabitation group) were conducted to determine the relative contributions of biophysical and biochemical CCMs on carbon fixation. NaH^13^CO_3_ was added as the sole carbon source in each group.

**Fig. 3:**
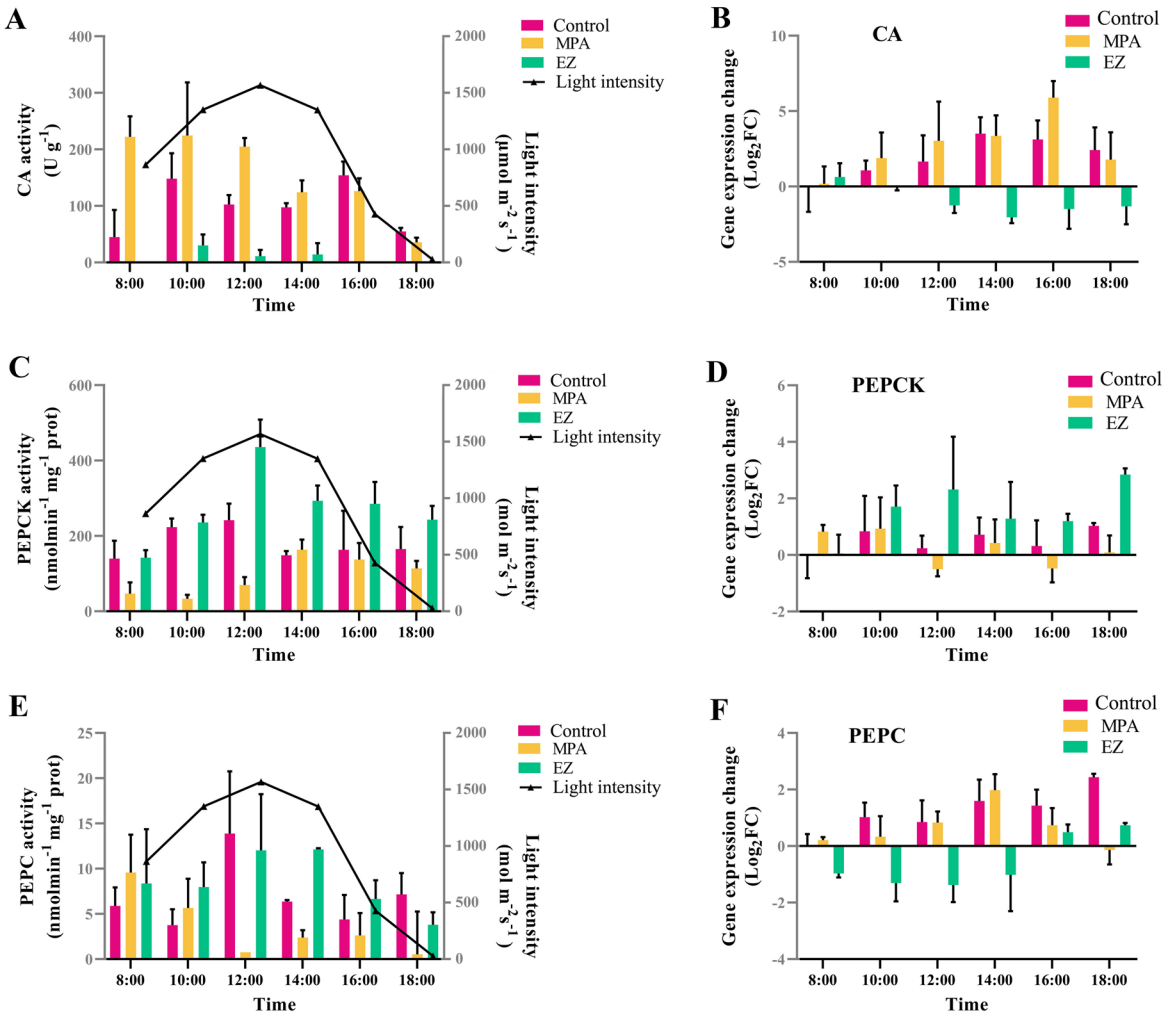
Diurnal patterns of CA, PEPCK, and PEPC activities and gene expression levels of *Ulva prolifera* in response to variations in light intensity in different treatment groups*.* **A** The pattern of CA activity. **B** CA relative gene expression. **C** The pattern of PEPCK activity. **D** PEPCK relative gene expression. **E** The pattern of PEPC activity. **F** PEPC relative gene expression. Gene expression is relative to the value measured in the control group at 8:00. *CA* carbonic anhydrase; *PEPCK* phosphoenolpyruvate carboxykinase; PEPC, phosphoenolpyruvate carboxylase

**Fig. 4:**
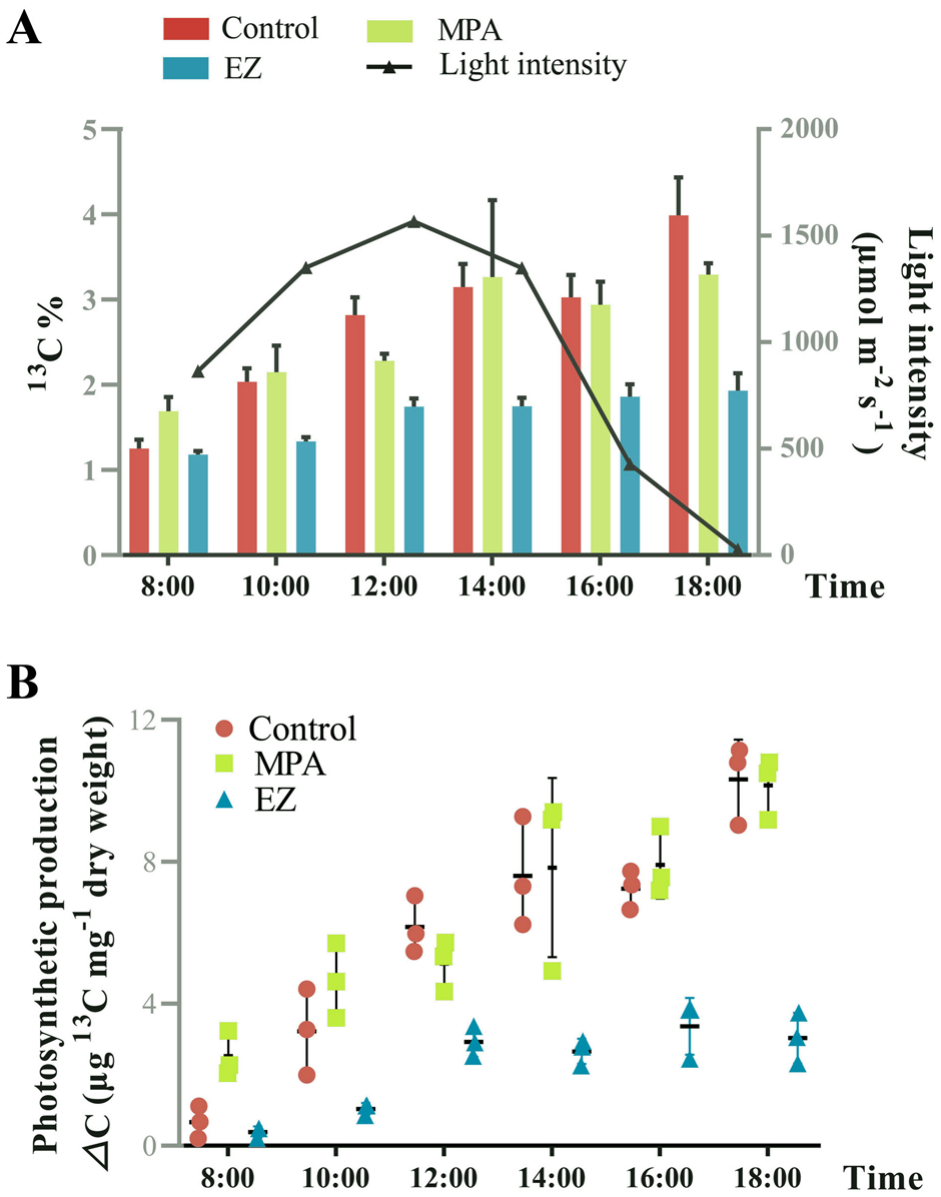
Diurnal variations of ^13^C % and photosynthetic production (*Δ*C) in *Ulva prolifera* subjected to different treatment. **A** Diurnal variations of ^13^C%. **B** Diurnal variations of *Δ*C. Each data is the mean of three replicate determinations. Bars are standard deviations of the mean data

**Fig. 5:**
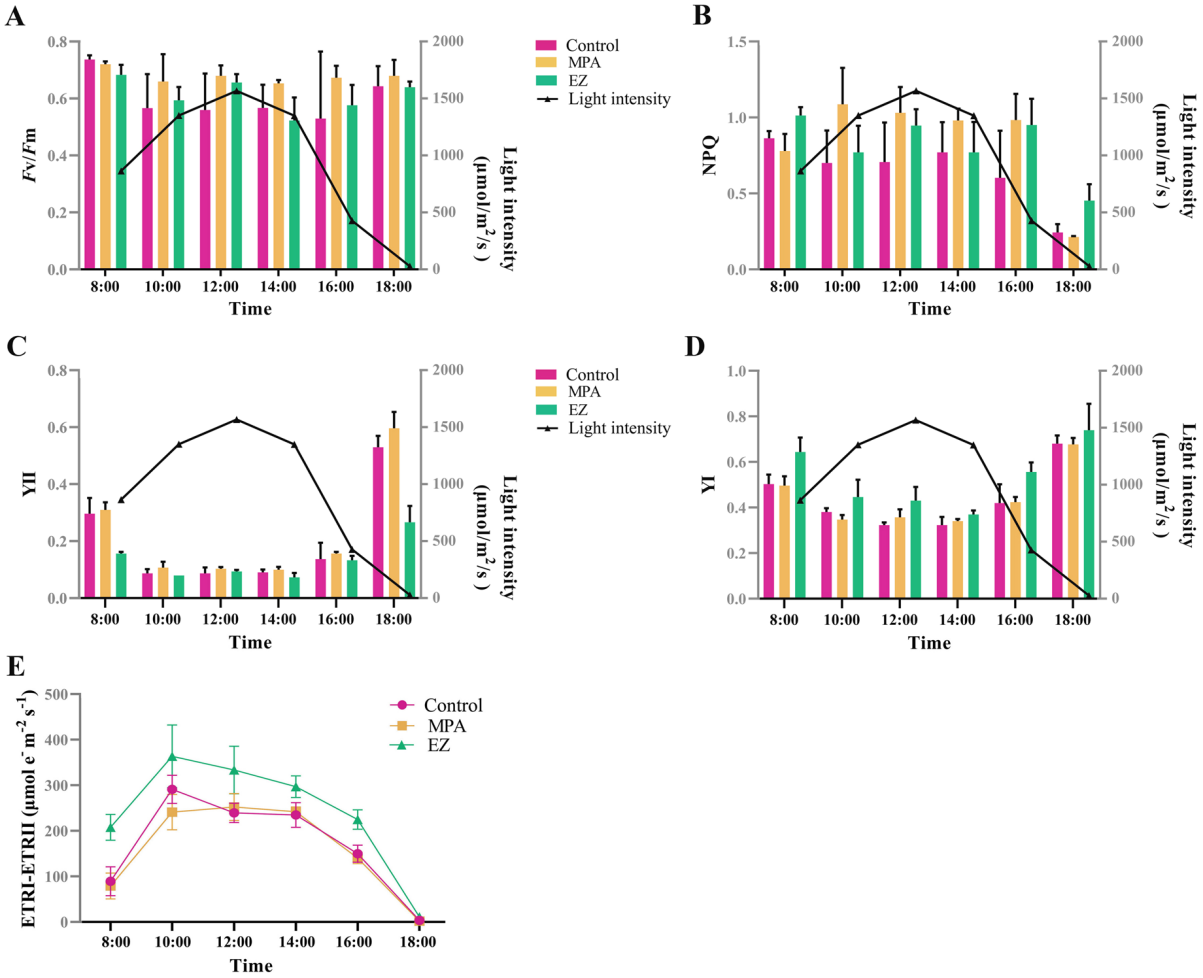
Changes of chlorophyll fluorescence parameters and ETRI–ETRII under diurnal variation irradiance. **A**. *F*v/*F*m. **B**. NPQ. **C**. YII. **D**. YI. **E**. ETRI–ETRII. Each data is the mean of three replicate determinations. Bars are standard deviations of the mean data. *Fv/Fm* PSII maximum photochemical yield, *NPQ* non-photochemical quenching, *YII* effective PSII quantum yield, *YI* effective PSI quantum yield

1. In the control group, CA activity was more active at 10:00 and 16:00 but decreased at high light intensity (12:00), and CA expression increased in the afternoon (Fig. 3A, B). PEPCK and PEPC activities were maximal at noon (12:00), while both PEPCK and PEPC expression increased during the course of the experiment (Fig. 3C–F). During the culture period, from 08:00 to 18:00, the ^13^C ratio and the photosynthetic ^13^C products gradually increased in the control group (Fig. 4A, B), probably as a result of the alternating action of biophysical and biochemical CCMs. The maximal photochemical efficiency of PSII (*F*v/*F*m) decreased under high light (Fig. 5A), whereas there were no significant changes in non-photochemical quenching (NPQ) except at 18:00 (Fig. 5B). With increasing light intensity, the actual conversion efficiency of light energy of PSII and PSI (YII and YI, respectively) decreased, while PSI- driven cyclic electron flow (CEF) activity increased (Fig. 5C–E).
2. In the EZ inhabitation group, CA activity was barely detectable, and CA expression significantly decreased from 10:00 (Fig. 3A, B), indicating that the biophysical CCM was inhibited in the EZ inhabitation group. PEPCK activity peaked at noon and was significantly higher than in the control group at 12:00 and 14:00 (*p* < 0.05), while PEPCK expression increased during the day (Fig. 3C, D). PEPC activity peaked under high light (12:00 and 14:00), and was significantly higher than the control group at 10:00 and 14:00 (*p* < 0.05) (Fig. 3E), but PEPC expression decreased during the experiment (Fig. 3F). These results indicate that the biochemical CCM was enhanced when the biophysical CCM was inhibited. The ^13^C ratio and the photosynthetic ^13^C products were significantly lower than the control group from 10:00 to 18:00 (*p* < 0.05), indicating that the carbon fixation was significantly inhibited in the EZ inhabitation group (Fig. 4A, B). *F*v/*F*m and NPQ in the EZ inhabitation show a similar trend to the control group (Fig. 5A, B). YI was significantly higher in the EZ inhabitation group than in the control groups at 12:00 (*p* < 0.05) (Fig. 5D), while the CEF of the EZ inhabitation group was significantly higher than that of the control group at all time points (*p* < 0.05) (Fig. 5E). These results indicate that PSI plays an important role in the CA-inhibited photosynthesis of *U. prolifera*.
3. In the MPA inhabitation group, the activities of PEPCK and PEPC were significantly inhibited compared to the control group at noon (12:00) (Fig. 3C, E), and the expression of PEPCK was inhibited from 12:00 to 18:00 (Fig. 3D), indicating that the biochemical CCM was inhibited in the MPA inhabitation group. However, CA activity was significantly improved from 8:00 to 14:00 compared to the control group (*p* < 0.05), along with the increased gene expression (Fig. 3A, B), indicating that the biophysical CCM was enhanced when the biochemical CCM was inhibited. The ^13^C ratio showed an increasing trend similar to that of the control group (Fig. 4A, B). The photosynthetic ^13^C products were significantly higher than those in the control group at 8:00 (*p* < 0.05), and then showed similar levels to the control group from 10:00 to 18:00 (*p* > 0.05) (Fig. 4B). The *F*v/*F*m of the MPA inhabitation group remained between 0.65 and 0.72 throughout the experiment, while the NPQ of the MPA inhabitation group showed an increasing trend under high irradiance (Fig. 5A, B). The trends of YII, YI and CEF were similar to those of the control group with no significant difference (Fig. 5C–E).

## Discussion

The results presented here demonstrate the contribution of biophysical and biochemical CCMs in providing CO_2_ for photosynthesis in *U. prolifera*. *U. prolifera* can utilize HCO_3_^−^ via the biophysical CCM at low CO_2_ concentration (Liu et al. 2020). In this study, the extracellular inhibitor AZ had no inhibitory effect on photosynthesis (*p* < 0.05), suggesting that HCO_3_^−^ uptake provides a carbon source for *Ulva* photosynthesis (Table 1) (Zuñiga-Rios et al. 2021), as in other *Ulva* species (Beer et al. 1990; Drechsler and Beer 1991). This work supports the hypothesis that biophysical CCMs are the major pathway in marine algae (Matsuda et al. 2017).

*U. prolifera* can perform C_4_ metabolism, as evidenced by identification of C_4_-related genes, substantial activities of key C_4_ enzymes, and δ^13^C photosynthetic products (Liu et al. 2020; Valiela et al. 2018; Xu et al. 2012). In this study, the role of C_4_ acid in photosynthetic carbon fixation of *U. prolifera* was further analyzed. C_4_ acids OAA or Asp stimulated O_2_ evolution in the absence of exogenous Ci (Fig. 1A), and C_4_ acid-driven photosynthetic O_2_ evolution was significantly reduced when PEPCK was inhibited by MPA (Fig. 1B), indicating that C_4_ acids could be decarboxylated to supply CO_2_ to *Ulva*. Addition of OAA inhibited Ci fixation by 55% when *Ulva* was photosynthesizing with sufficient NaH^13^CO_3_ (2 mmol/L) (Table 2), suggesting that OAA could provide a large fraction of photosynthetically fixed carbon to *U. prolifera.* The OAA-dependent O_2_ evolution rate was modulated by the CO_2_ concentration in the cultured thalli environment (Table 3), indicating that C_4_-dependent carbon fixation is an important mechanism to concentrate CO_2_ in *U. prolifera*. These results confirm the important role of biochemical CCM in photosynthetic carbon fixation of *U. prolifera*.

**Fig. 6:**
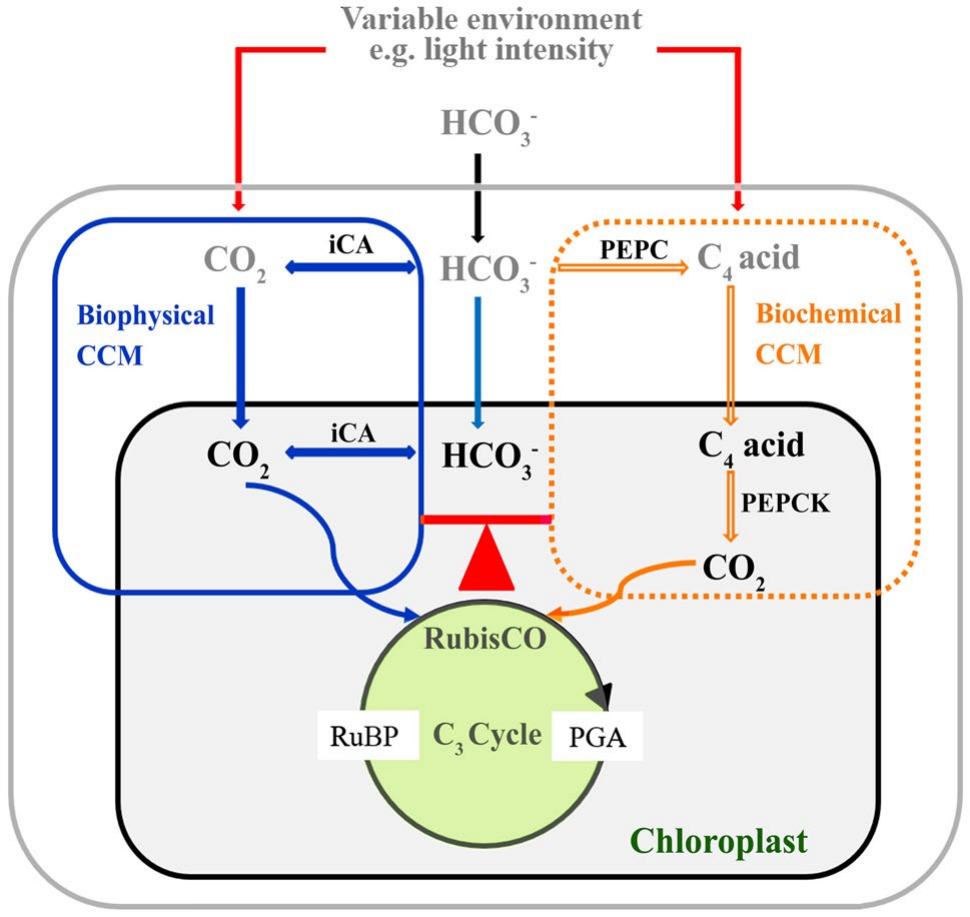
Schematic view of CO_2_ accumulation processes in *U. prolifera*. The model incorporates a biophysical CCM based on the conversion of inorganic carbon and a biochemical CCM based on the formation of C_4_ acids. In the biophysical CCM, HCO_3_^−^ is converted to CO_2_ by iCA, which plays a dominant role in carbon fixation of *U. prolifera* (shown in blue). In the biochemical CCM, HCO_3_^−^ is first fixed to C_4_ acid and then decarboxylated to produce CO_2_ for RubisCO, which plays a supporting role in carbon fixation of *U. prolifera* (shown in yellow). The activities of the biophysical and biochemical CCMs are influenced by environmental factors, and the two CCMs complement each other to provide CO_2_ for carbon fixation in *U. prolifera* under different environmental conditions*.* Abbreviations: *RubisCO* ribulose-1,5-bisphosphate, *iCA* intracellular carbonic anhydrases, *PEPC* phosphoenolpyruvate carboxylase, *PEPCK* phosphoenolpyruvate carboxykinase, *RuBP* ribulose-1,5-bisphosphate, *PGA* 3-phosphoglycerate

Previous studies have shown that environmental factors regulated not only key C_4_ enzymes but also CA activities. For example, Wang et al (2020) reported that CA expression was inhibited by high irradiance, while high irradiance could induce PEPC and PEPCK activities in *U. prolifera* (Liu et al. 2020), which was also found in this study (Fig. 2A, B). These results indicate that two CCMs could coordinate their roles to realize complementation based on changing environmental conditions (Fig. 6). In this study, when the intracellular CA was inhibited by EZ, the significant increase of PEPC and PEPCK activities showed that C_4_ acids (5 mmol/L OAA or Asp) could restore the photosynthetic O_2_ evolution of *U. prolifera* (Figs. 1C, 2C, D) and enhance the biochemical CCM. Notably, PEPCK activity was significantly increased after the EZ treatment, but there was no significant difference between PEPCK activity after 2 h of EZ treatment and that after 1 h of EZ treatment (*p* < 0.05) (Fig. 2C), which may be due to the different demand for PEPCK activity resulting from the different degree of inhibition by EZ. PEPC activity after 4 h of EZ treatment was decreased compared to 2 h of EZ treatment (Fig. 2D), probably influenced by the concentration of C_4_ acid in the cells. These results suggest that the biochemical CCM’s activity is affected by a range of factors, such as C_4_ acid concentration.

The relative contributions of biophysical and biochemical CCMs under fluctuating sunlight were further estimated. Addition of the PEPCK inhibitor MPA suppressed the activities and transcription of the key C_4_ enzymes, PEPC and PEPCK (Fig. 3C–F). The exception is that PEPC expression increased in the MPA inhabitation group at several time points, suggesting that there may be multiple genes encoding PEPC in *U. prolifera*. Photosynthetic ^13^C products in the MPA inhabitation group were significantly higher than those in the control group at 8:00, and the corresponding CA activity in the MPA inhabitation group increased significantly (Figs. 3A, 4B), implying that the biophysical CCM was enhanced in the MPA inhabitation group to promote photosynthetic carbon fixation. Further analysis showed that photosynthetic ^13^C products in the MPA inhabitation group could be supplemented to the control level even when key C_4_ enzymes were inhibited during the high light period (from 10:00 to 14:00) (Figs. 3C–F, 4B), indicating that the biophysical CCM can compensate for the lack of biochemical CCM in *U. prolifera*. This result is consistent with the high quantum yield (*F*v/*F*m) (Fig. 5A) and the ability to dissipate excess light energy (NPQ) (Fig. 5B) under high light conditions in the MPA inhabitation group. *U. prolifera* could dissipate excess light energy through other pathways and provide energy for the biophysical CCM when the C_4_ pathway was inhibited. Burlacot et al (2022) found that CEF and pseudo-cyclic electron flow (pseudo-CEF) can provide energy for CCM in the green alga *Chlamydomonas*. In this study, CEF increased with increasing irradiance in the MPA inhabitation group, which was similar to that in the control group (Fig. 5E). It is speculated that *U. prolifera* might use CEF or pseudo-CEF, or both, to drive the biophysical CCM process that promotes carbon fixation under the joint action of high CA enzyme activity. Thus, *U. prolifera* could promote carbon fixation through the synergy between energy and biophysical CCM essential elements in the MPA inhabitation group.

The expression and activity of CA were significantly suppressed in the EZ inhabitation group during the experiment (Fig. 3A, B), suggesting that the biophysical CCM was inhibited in the EZ inhabitation group. As discussed above, if the biochemical CCM can compensate for the lack of a biophysical CCM, then a large amount of energy and the cooperation of components in the C_4_ mechanism is required. Gu et al (2022) reported that *U. prolifera* could provide energy for the biochemical CCM by increasing CEF. In this study, CEF in the EZ inhabitation group was significantly higher than that in the control group (Fig. 5E), indicating that CEF could provide energy for the operation of biochemical CCM in the EZ inhabitation group. In addition, results presented here show that the PEPC and PEPCK enzyme activities were higher in the EZ inhabitation group than in the control group, indicating that the biochemical CCM was active in the EZ inhabitation group (Fig. 3C, E). The photosynthetic ^13^C products of the EZ inhabitation group increased but were significantly lower than those of the control group (Fig. 4B), indicating that the biochemical CCM could not fully compensate the role of biophysical CCM in carbon fixation of *U. prolifera*. This may be due to the fact that C_4_ acid can be used for functions other than as a carbon source (Salvucci and Bows 1983). It was also consistent with the finding that high concentrations of C_4_ acid could restore, but low concentrations of C_4_ acid failed to restore, photosynthetic O_2_ evolution when the biophysical CCM was inhibited by EZ (Fig. 1C). At noon, when the C_4_ mechanism was most active, the photosynthetic ^13^C products in the EZ inhabitation group were approximately 47% of those in the control group (Fig. 4B). Combined with the results of C_4_-Ci competition experiment, OAA can provide 55% of the carbon fixation, it is speculated that the biochemical CCM accounted for the total carbon fixation ~ 50% under high light condition. The C_4_-based system may be a complement to the biophysical CCM and is of great ecological significance for *Ulva* under adverse circumstances.

In conclusion, our results demonstrate that *U. prolifera* can absorb HCO_3_^−^ and has two efficient CCMs, biophysical and biochemical CCMs, where biophysical CCM plays a dominant role in carbon fixation and biochemical CCM plays a complementary role under fluctuating light conditions (Fig. 6). Therefore, the formation of floating blooms of *U. prolifera* can benefit from the cooperation of the biophysical and biochemical CCMs under harsh environmental conditions of sea surface.

## Supplementary Information

Below is the link to the electronic supplementary material.

Supplementary file1 (DOCX 16 KB)

## Data Availability

Data will be made available on request.
