# Supplementary material for: The contribution of biophysical and biochemical CO2 concentration mechanisms to the carbon fixation of the green macroalga Ulva prolifera
Source: Mar Life Sci Technol. 2024 Dec 12;7(3):537–48. doi: 10.1007/s42995-024-00265-7 (PMC12413351; doi:10.1007/s42995-024-00265-7)
Supplement: Supplementary file 1 — Supplementary file1 (DOCX 16 KB) [file 42995_2024_265_MOESM1_ESM.docx]

**Marine Life Science & Technology**

**The** **contribution of biophysical and biochemical CO_2_ concentration mechanisms in the carbon fixation of** **the green macroalga *Ulva prolifera***

**Xiaohua Zhang^1^, Guang Gao^2^, Zhengquan Gao^1^,** **Kunshan Gao^2^,** **Dongyan Liu^3^***

1 School of Pharmacy, Binzhou Medical University, Yantai 264003, China

2 State Key Laboratory of Marine Environmental Science, Xiamen University (Xiang'an Campus), Xiamen 361102, China

3 State Key Laboratory of Estuarine and Coastal Research, East China Normal University, Shanghai 200241, China

*Corresponding authors: E-mail address: [dyliu@sklec.ecnu.edu.cn](mailto:dyliu@sklec.ecnu.edu.cn)

**Supplementary Table S1** Primers used for RT-qPCR

| Gene Name | Primer Sequence (5'-3')  Forward/Reverse |
| --- | --- |
| Carbonic anhydrase (CA) | GTGACGTCGTTCAACATGCC  CAGGTCAAACTGCAGAGGGT |
| phosphoenolpyruvate carboxylase kinase (PEPCK) | GCTTGCAGACGCCGAATATG  TGGATCGTGGCTGCAAGATT |
| phosphoenolpyruvate carboxylase (PEPC) | GGCGCGTACGTCATCTCG  CTCGAACAGCGGCACCA |
| 18S rDNA | ATTAGATACCGTCGTAGTCTCAACC  TCTGTCAATCCTTCCTATGTCTGG |
